# Nuclear Hormone Receptors and Their Ligands: Metabolites in Control of Transcription

**DOI:** 10.3390/cells9122606

**Published:** 2020-12-04

**Authors:** Lian Jing Tao, Dong Eun Seo, Benjamin Jackson, Natalia B. Ivanova, Fabio Rinaldo Santori

**Affiliations:** 1 Department of Genetics, Center for Molecular Medicine, University of Georgia, Athens, GA 30602, USA; taojinglian@uga.edu (L.J.T.); dongeun.seo@uga.edu (D.E.S.); benjamin.jackson1@uga.edu (B.J.); 2 Department of Immunobiology, Yale University, New Haven, CT 06520, USA

**Keywords:** nuclear hormone receptors, ligands, terpenoid, fatty acid, thyroxine, porphyrins, history, evolution

## Abstract

Nuclear hormone receptors are a family of transcription factors regulated by small molecules derived from the endogenous metabolism or diet. There are forty-eight nuclear hormone receptors in the human genome, twenty of which are still orphans. In this review, we make a brief historical journey from the first observations by Berthold in 1849 to the era of orphan receptors that began with the sequencing of the *Caenorhabditis elegans* genome in 1998. We discuss the evolution of nuclear hormone receptors and the putative ancestral ligands as well as how the ligand universe has expanded over time. This leads us to define four classes of metabolites—fatty acids, terpenoids, porphyrins and amino acid derivatives—that generate all known ligands for nuclear hormone receptors. We conclude by discussing the ongoing efforts to identify new classes of ligands for orphan receptors.

## 1. Introduction

As a family, nuclear hormone receptors (NHRs) represent some of the most biologically important transcription factors that integrate cellular metabolism and function. NHR activities are controlled by binding to small molecules or ligands derived from endogenous metabolism, hormones or vitamins obtained from the diet [1]. A typical NHR contains a DNA binding domain, which recognizes a specific DNA motif, and a ligand binding domain, which regulates the NHR activity. In the absence of the ligand, the ligand binding domain may adopt either an inactive or repressive conformation [2]. Upon ligand binding, the resulting conformational changes allow recruitment of co-activators and induction of target gene expression on promoters containing a positive hormone receptor element or target gene repression on promoters containing a negative hormone receptor element [3]. The ability to be regulated by small molecules makes these receptors ideal targets for drug discovery: 16% of all drugs target NHRs [4].

Metabolites are widely used for intercellular communication in both prokaryotes and eukaryotes [5]. For example, fatty acids are dedicated to “quorum sensing” in bacteria, whereas peptides, cyclic AMP, and lysophosphatidic acid mediate a variety of responses in eukaryotes [5]. Two common metabolite signaling systems, G-protein coupled receptors (GPCRs) and cAMP receptors, are abundant throughout the animal and plant kingdoms [5]. In contrast, NHRs are observed exclusively in animals and were first detected in sponges [6].

Identification of ligands for NHRs is fundamental for understanding how these transcription factors function. In this review, we focus on metabolic pathways that produce ligands for NHRs and the mechanisms by which NHR ligands mediate intercellular communication.

## 2. A Brief History of NHRs and Their Ligands

### 2.1. Phenomenology (1849–1914)

It has been known since antiquity that eunuchs are passive, non-aggressive and sexually uninterested in females. The mechanisms underlying this phenotype were not well understood until 1849, when Berthold conducted an experiment that marked the beginning of endocrinology. He removed the testicles of roosters and observed that these animals, like eunuchs, became passive and non-aggressive. The roosters also had underdeveloped vocalization, combs and neck lobes [7]. Most importantly, when Berthold transplanted testicles back to the castrated animals, secondary sexual characteristics were restored, even though the transplanted testicles were in ectopic sites and had their normal innervation removed [7]. Forty years later, Brown-Sequard demonstrated that injection of testicular extract could increase aggression and stamina in male animals, including man [8]. It was at that time proposed that testicular extract could be used as rejuvenation therapy [8]. Brown-Sequard’s experiments clearly suggested that it was a substance in the testicles that could promote this effect, and there was no need for the organ or cells themselves. Less dramatic, but equally significant, were related observations on the thyroid gland. Physicians had observed that surgical removal of the thyroid gland resulted in myxedema and that injection of thyroid extract was sufficient to restore thyroid function of myxedema patients [9]. In 1896, Bauman demonstrated that a peptidic compound rich in iodine, “thyroiodin”, could be isolated from the thyroid extract and used to treat myxedema in animals [10]. Thus, whole organs could be replaced by extracts, suggesting that a messenger substance mediates the function of these glands.

To unify these observations, Starling created the concept of “hormones” that are released by one organ but affect other distant organs. The term “hormone” was derived from the Greek for “I excite” or “arouse” [11]. Starling extended the list of endocrine organs suspected of producing hormonal substances to include the adrenal glands, pancreas and ovary, in addition to the thyroid and the testes [11,12]. The best examples included the role of the supra-renal gland in the control of blood pressure [11], the relationship of the pancreas and diabetes [11], the role of the thyroid in the development of the nervous system and metabolism [12], the control of secondary sexual characteristics by the testes and ovaries [12] and the influence of ovaries on pregnancy [12]. Thus, the concept of hormones brought multiple phenomenological observations into the sphere of one concept that could be tested experimentally.

### 2.2. Hunting for Hormones (1915–1984)

Immediately, the hunt for hormones began. Starling had reinforced previous observations that the factor present in the thyroid was stable, since the function of the thyroid could be replenished by providing thyroid extract in the diet [12]. Taking this lead, Kendal isolated the active component of the thyroid gland, thyroxine [13]. Bioactive thyroxine that could replace “natural thyroid extract” was synthesized in 1927 by Harington and Barger [14]. Kendal’s discovery of thyroxine in 1915 marks the second phase in the history of NHRs: the era of orphan ligands. After this discovery, a quest for the identification, characterization and synthesis of hormones resulted in the discovery and synthesis of many hormones including estrogens [15,16], androgens [17,18,19], progesterone [20] and corticoids [21,22,23]. The isolation and purification of hormones was a heroic phase in biochemical research. For example, the isolation of the first androgen required the purification of 15 mg of pure steroid from an estimated 25,000 L of human male urine [24].

### 2.3. Ligands Meet Receptors (1985–1997)

**Figure 1:**
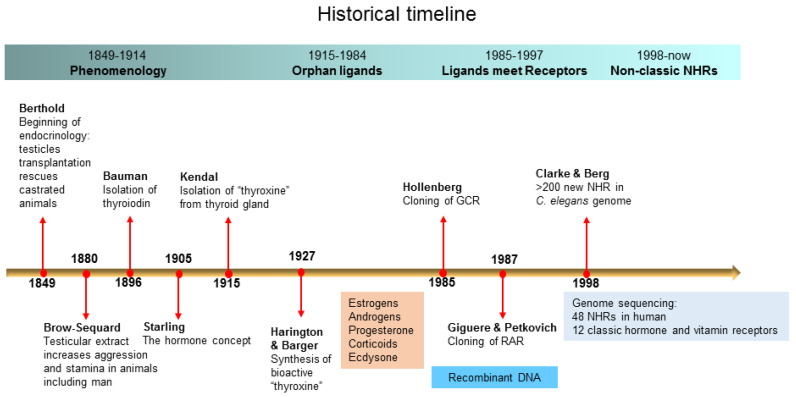
A brief history of nuclear hormone receptors (NHRs). Shown are the landmark moments in the history of NHRs.

NHR ligands remained orphans until the advent of recombinant DNA technology. The first NHR to be identified was the glucocorticoid receptor (GCR). Here, the ligand, glucocorticoid, was used to purify the receptor [25,26] which was then used to generate GCR-specific monoclonal antibodies to screen bacterial protein expression libraries to identify a GCR cDNA [27]. Identification and sequencing of GCR enabled the discovery of the retinoic acid receptor (RAR) and other members of the NHR family via homology-based cDNA library screening [28,29]. Thus, many orphan ligands were quickly “adopted” by their receptors [1]. The history of NHRs is summarized in Figure 1.

### 2.4. Orphan NHRs Meet Their Ligands (1998–Present)

**Figure 2:**
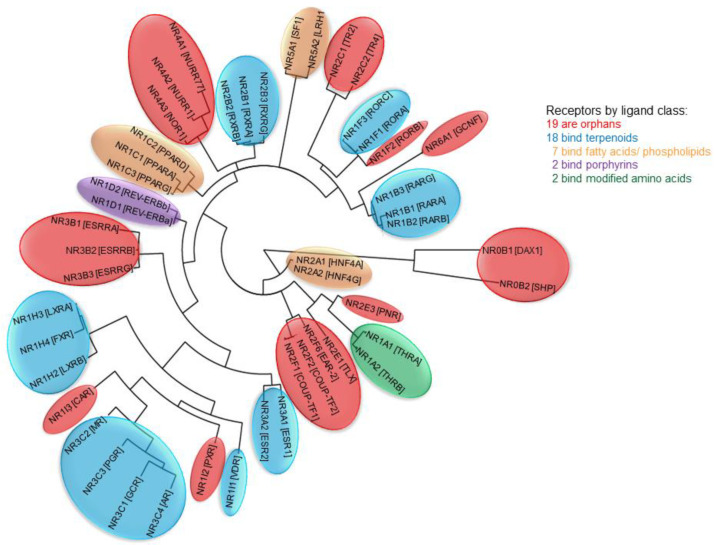
The 48 human NHRs and their ligands. Nuclear hormone receptors are defined by their homology to the steroid receptors [38]. This feature distinguishes the NHRs from other nuclear receptors regulated by small molecules such as the Per-Arnt-Sim (PAS) superfamily of transcription factors [39]. A dendrogram of the human nuclear hormone receptor family was generated using phylogeny.fr [40]. Color-coded balloons are used to label the ligand class for each individual NHR.

Genome sequencing has provided us with a finite number of NHRs (Figure 2). Humans, for example, have 48 NHRs, only 12 of which are classic hormone and vitamin receptors [1]. Other organisms, like *C. elegans*, have >200 NHRs in their genome [30]. The majority of NHRs are orphan receptors. Over the years, many orphan NHRs have been deorphanized. Examples include the liver X receptors (LXRs) LXRα and LXRβ, which bind oxysterols [31]; farnesoid X receptor (FXR), a receptor for bile acids [32,33]; the retinoic acid-related orphan receptors (RORs) RORα and RORγ that bind cholesterol biosynthetic intermediates [34]. These success stories also apply to orphan nuclear hormone receptors from other species such as Daf12 of *C. elegans*, which binds dafachronic acids [35]. Identification of ligands for NHRs in nematodes and insects could be of importance for the treatment of diseases caused by parasitic nematodes in man [36] and pest control in agriculture [37]. However, despite these early discoveries, almost half of human NHRs and most NHRs from other species are still orphans. 

## 3. Metabolome as a Source of NHR Ligands

The mammalian metabolome is composed of endogenous and exogenous metabolites. The endogenous metabolome contains all metabolic products produced by the organism itself. For example, cholesterol is an endogenous metabolite synthesized by mammalian cells from the two-carbon acetate group of acetyl-CoA [41]. The endogenous metabolome in mammals is estimated to contain approximately 1500 backbones/compounds, which are further modified to generate an enormous diversity of isomers [42]. The exogenous metabolome contains diet-derived as well as synthetic compounds that are modified by the enzymes of the organism. All degraded drugs are part of the exogenous metabolome, which is estimated to be at least ten to one hundred times larger than its endogenous counterpart [42].

**Figure 3:**
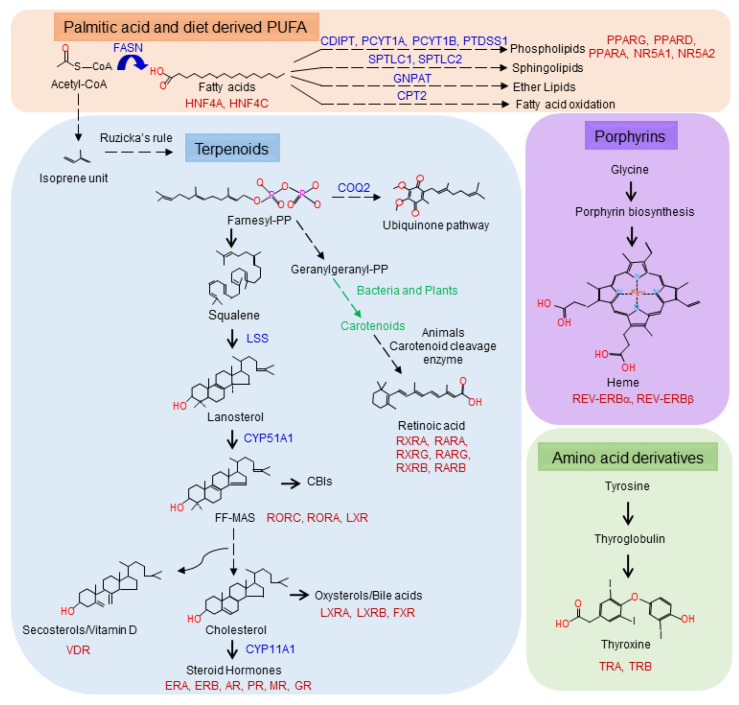
The biosynthetic routes for the known sources of human NHR ligands. Fatty acids (orange) and terpenoids (blue) are generated from acetyl-CoA. Fatty acids can be further modified into phospholipids, glycerolypids, sphingolipids, ether lipids and fatty acid oxidation products. Acetyl-CoA is used to generate the isoprene units for terpenoid biosynthesis. Mammals will generate farnesyl and geranylgeranyl pyrophosphate, dolichol (not shown), ubiquinone and cholesterol. Bacteria and plants utilize geranylgeranyl pyrophosphate to generate carotenoids that can be used by animals to generate retinoic acid. Porphyrins are synthesized from glycine and thyroid hormones from tyrosine.

NHRs are found in the most primitive animals, such as the demosponge *Amphimedon queenslandica* [43]. Sponges have two NHR-like proteins: AqNR1 and AqNR2. AqNR2 is the ortholog of the mammalian HNF4a/NR2A family [6], while AqNR1 is the ancestral receptor for all other NHRs. HNF4a/NR2A1 binds fatty acyl-CoA [44], and indeed fatty acids induce AqNR1 and AqNR2 transactivation [6], suggesting that one of the first ligands of ancestral NHRs was a fatty acid derivative. After the sponges, there was an expansion of the NHR superfamily, and the common ancestor that gave origin to vertebrates had receptors representative of the NHR families NR2C, NR5A1 (SF-1), NR6A1(GCNF), RXRs, ERRs and steroid receptors [6]. Here, we already see a branching of potential ligands: NR5A1 (SF-1) binds phosphatidylinositol [45], a fatty acid conjugated to inositol; RXRs bind 9-cis-retinoic acid [46,47], a retinoid; the steroid hormone receptors and estrogen-related receptors (ERRs) bind steroid hormones (reviewed in [1]). The ligands now include modified fatty acids and terpenes (Figure 2 and Figure 3). A second expansion of ligands recognized by NHRs occurred in the common ancestor of the bilaterians, before the branching of the protostomes and deuterostomes [6]. We now see the inclusion of additional ligand families represented by the thyroid hormone receptor that binds thyroxine [48,49], and REV-ERBα (NR1D1) and REV-ERBβ (NR1D2), which bind heme, a porphyrin [50]. All these ligands are derived from four main classes of metabolites: fatty acids, terpenoids, porphyrins and modified amino acids (Figure 2 and Figure 3).

A common thread between these ligands is the presence of a hydrophobic backbone with a head group that is either charged or capable of forming van der Walls or hydrogen bonds with the receptor. After the bilaterians, the expansion of the NHR superfamily was not followed by an expansion of recognized ligand classes. Rather, receptors recognize other derivatives of the four main classes of metabolites described above. For example, PPARγ binds to modified fatty acids, hydroxylated polyunsaturated fatty acids and prostaglandins [51,52,53], whereas FXR binds bile acids [32,33], which are modified terpenes.

### 3.1. Fatty Acid Family Ligands

Fatty acids are the ancestral family of ligands for NHRs, and they include components derived from the endogenous metabolism as well as diet-derived essential fatty acids such as linoleic, linolenic and arachidonic acids. Fatty acids are a class of diverse ligands. According to the lipidmaps database [54], there are currently 9985 fatty acids. Fatty acids can be modified at the hydrophobic tail (saturation, desaturation) and/or head group (glycerol, choline, ethanolamine, amino acid or carbohydrates). This greatly increases the diversity of this class of ligands. So far, there are 22,471 known glycerolipid, glycerophospholipid and sphingolipid derivatives of fatty acids (https://www.lipidmaps.org/resources/databases/index.php?tab=lmsd).

### 3.2. Terpenoid Family Ligands

Most ligands for nuclear hormone receptors come from the terpenoid family (Figure 2). Terpenoids are a class of natural compounds that includes over 80,000 known molecular species [48] and are easily modified by monooxygenation reactions catalyzed by CYP450 enzymes that increase diversity even further [55]. Most terpenoids are derivatives of 5-carbon isoprene units following what is known as Ruzicka’s rule [56]. In mammalian cells, terpenoids are synthesized from acetate [41] through the mevalonate [57] pathway. Terpenoids include all sterol lipids [41,56] and retinoids such as vitamin A and its precursor, β-carotene, which is generated in bacteria, fungi and plants [58]. Of particular interest are the sterol lipids. During evolution, there was an “explosion” of receptors that recognized sterol-type structures in the common ancestor of the bilaterians [6]. In humans, 12 NHRs with known ligands are activated by sterol lipids, including the receptors for estrogen [59,60], progesterone [61,62], testosterone [63,64], mineralocorticoids [65], glucocorticoids [27], oxysterols (LXRα and β) [31], bile acids (FXR) [32,33], cholesterol biosynthetic intermediates RORα and RORγ [34] and, for secosteroids, the vitamin D receptor (VDR) [66]. The diversity of sterol lipids is generated via modification of a basic backbone with four rings, A, B, C and D (Figure 3), by addition or removal of double bonds, hydroxyl and keto groups as well as different isomer patterns [67]. This diversity of modifications generates new compounds, distinct enough to be classified as subclasses of steroids. For example, in secosteroids, vitamin D and its related compounds are formed by the opening of the B ring in the sterol backbone [67]. Similarly, estrogens contain three double bonds in ring A [67]. Thus, receptors that initially recognized one sterol lipid could have been co-opted during evolution to act as a receptor for the new class of steroids, leading to the observed expansion in sterol lipid NHRs.

### 3.3. Porphyrins

The next class of compounds that generate ligands for NHRs is the porphyrins. These are lipophilic metabolites in which the porphyrin backbone is associated with a metal atom, such as Mg for chlorophyll or iron for heme. NHRs such as E75 in *Drosophila melanogaster* [68] or REV-ERBα (NR1D1) and REV-ERBαβ (NR1D2) in vertebrates bind heme [50,69]. In the case of E75, heme is a structural component of the receptors that is modified to serve as a sensor for diatomic gases like carbon monoxide and nitric oxide [68]. In contrast, in mammalian cells NR1D1 and NR1D2 function as direct heme sensors [50,69]. The finding of insect NHRs that sense gas-modified heme increases the diversity of potential NHR ligands.

### 3.4. Amino Acid Derivatives

Amino acids were the first metabolites identified as a source of ligands for NHRs. For example, thyroxine (T4) and the active derivative triiodothyronine (T3) are both derived from tyrosine and synthesized from a protein precursor, thyroglobulin, making it a protein/peptide-derived ligand (reviewed in [70]). In these cases, a dedicated organ, the endostyle in protochordates and the thyroid gland in jawed vertebrates, evolved to produce an iodinated amino acid derivative that has no other function in the organism but to act as a hormone [71]. Thyroid hormone-like substances are also found in other invertebrates, but their functions are still poorly understood. Interestingly, thyroid hormone-like substances are produced by marine algae (Diatoms), suggesting that these substances originally acted like vitamins for plankton-feeding organisms [71]. In accord with this hypothesis, the thyroid gland could have evolved exclusively to generate thyroid hormone-like substances in animals that do not feed on plankton [71]. Thus, a vitamin became a hormone. No other NHRs so far have been identified that recognize amino acid derivatives.

## 4. Orphan Receptors, What Ligands?

It has been suggested that some orphan NHRs are ligand-independent or constitutively active. A good example is NR4A1, an NHR with strong transcriptional activity in most mammalian cells. The main argument for NR4A1′s ligand-independent function is a crystal structure showing that the ligand-binding pocket of NR4A1 is too small to accommodate a ligand [72]. However, such evidence must be taken with a grain of salt. Initially, the crystal structure of REV-ERBβ identified a small ligand-binding pocket filled with bulky hydrophobic amino acid residues [73]. However, further studies showed that REV-ERBβ binds heme [50]. Another argument, given the conservation of the ligand backbones, is that orphan NHRs bind the same classes of ligands as non-orphan receptors. However, this focus on known ligands could be misleading. It is possible that we have not yet discovered all the molecules that affect our physiology.

What is the possibility that new vitamins could be found? Mice can be maintained on a chemically defined low molecular weight diet for several generations [74]. Similarly, *Drosophila melanogaster* can be reared in chemically defined conditions [75]. These results seemingly argue against the idea that there are unknown vitamins “out there”. On the other hand, many of these diets have undefined components extracted from vegetable sources that could introduce a contaminant into the system. The dramatic effect that such contaminants may have on development is illustrated by the example of *C. elegans*. Many nematodes are cholesterol auxotrophic [76]. and *C. elegans* can develop normally in chemically defined agar plates. However, when the sterol content of the agar is removed by extraction with chloroform and methanol, there is severe impairment of worm development [77]. Addition of the proper cholesterol enantiomer can completely restore *C. elegans* development in agar plates where all lipids are extracted with organic solvent [77]. Later, cholesterol was shown to be a precursor for dafachronic acid, which is a ligand for Daf12 [35], an NHR that is essential for C. elegans development.

One way to address whether there are exogenous components to NHR function is by testing NHR transcriptional activity in different cell lines in chemically defined medium [34]. For example, most cholesterol-sufficient mammalian cells, such as HEK293 or HeLa cells, show strong RORγ transcriptional activity [34]. In contrast, mammalian cell lines with genetic deletion in the cholesterol biosynthetic pathway and cells derived from cholesterol auxotroph, such as insect cells S2 and Kc167, had a reduction or a complete block in RORγ transcriptional activity [34]. Tissue culture medium is prepared from microbial sources, and there is little contamination with eukaryotic metabolites. An in-house, chemically defined medium can be developed with basal media such as DMEM, RPMI or Grace’s medium supplemented with insulin, transferrin and Pluronic F68 as a replacement for bovine serum albumin, warranting that there are no mammalian- or plant-derived molecules in the medium [34]. Detection of NHR activity in specific cell lines in a chemically defined medium is a good indicator that the ligand is endogenously produced or modified by the cells themselves. One can now identify the ligand by genetic and chemical means to certify whether it is a member of a known ligand family or an entirely new class of ligand.

What about new hormones produced by tissues or specific cells in the organism? This question can be addressed in the same manner as we addressed the possibility of new vitamins. Hormones are “messengers” generated by one cell as a communication component to another cell/organ. Such communication systems could act within the cell (autocrine fashion), on an adjacent cell (paracrine fashion) or on a distantly located cell (endocrine fashion). We would expect that hormone-like molecules should be produced by a restricted number of cell lineages.

New hormone-like molecules could come from many sources. One is intermediates in biosynthetic pathways. For example, 4α-carboxy-zymosterol and other biosynthetic intermediates with a double bond at carbon 8 have been shown to be ligands for RORα and RORγ [34]. Desmosterol, another intermediate of cholesterol biosynthesis, can also function as a ligand for RORγ [78] and LXR [79]. Another intermediate, 7-dehydrocholesterol, is a precursor for vitamin D (reviewed in [1]). Interestingly, some cholesterol biosynthetic intermediates accumulate in tissues. For example, FF-MAS accumulates in ovaries and T-MAS accumulates in the testis, where they promote meiosis [80]. Similarly, metabolic products of the conversion of lanosterol into FF-MAS, such as the 3β-lanost-32-aldehydes, can accumulate in cells [81,82]. The mechanism by which these sterols act is still unclear. However, it is tempting to speculate that these metabolites may act through NHRs via autocrine or paracrine mechanisms. Indeed, FF-MAS has been shown to activate LXR and RORγ in reporter assays [31,34], and lanosterol aldehydes have been suggested as candidate RORγ ligands [34]. It remains to be seen whether such intermediates or their derivatives could also have endocrine functions.

Sterol lipids are not the only pathways producing metabolites that could be used as hormone-like substances. Sphingolipids generally have a head group derived from serine. However, the preference for serine is dictated in cells by the availability of serine and alanine; at low serine, high alanine concentrations there is production of sphingolipids with alanine head-groups (1-deoxysphingosines) [83]. 1-deoxysphingosines lack the C1 hydroxyl group of serine-based sphingolipids, and they cannot serve as precursors for the synthesis of phospho- or glycosphingolipids or be degraded by the known sphingolipid catabolic pathways [84]. These are only a few examples of possible hormone-like compounds. Lipidomics studies have identified a large number of new lipids with unknown function [54], and some of these could possess hormone-like activities.

## 5. Common Properties of NHR Ligand Biosynthetic Pathways

The common feature of NHR ligands is a hydrophobic backbone associated with chemical groups that allow for the formation of hydrogen bonds or van der Waals interactions, for example the carboxy group of fatty acids and bile acids. There has been no report of a totally hydrophobic compound as an NHR ligand. The main sources of endogenous metabolites with hydrophobic backbones attached to chemical groups that allow for hydrogen bonding are the fatty acid and the terpenoid pathways. It is possible that the preference for these pathways may be an evolutionary accident since fatty acid derivatives and sterol lipids were the first ligands for the ancestral receptors [6]. Alternatively, these backbones could have been selected by chance, since the fatty acid and terpenoid pathways are also the most diverse groups of metabolites. Generally, the compounds that serve as the sources of ligands for NHRs are targeted by many processing enzymes. This includes enzymes that process polyunsaturated fatty acids into prostaglandins, resolvins and maresins [85,86], as well as the monooxygenases and dehydrogenases that process sterol lipids into steroid hormones, vitamin D and bile acids.

The majority of known NHR ligands are soluble metabolites that would allow for autocrine, paracrine or endocrine functions. There are a few notable exceptions: the ligand for PPARα is a phosphatidylcholine, a structural component of the membrane [87]. However, the jury is still out on PPARα, as other potential ligands have been suggested such as Coenzyme Q10 [88], oleylethanolamine [89] or 7-hydroxydocosahexaenoic acid [90]. Oleylethanolamine and 7-hydroxydocosahexaenoic acid are the most exciting findings, since they may play hormone-like or vitamin-like roles that fit with the standard properties we suggest for NHR ligands.
